# What’s in a Click? The Efficacy of Conditioned Reinforcement in Applied Animal Training: A Systematic Review and Meta-Analysis

**DOI:** 10.3390/ani10101757

**Published:** 2020-09-28

**Authors:** Nicole Pfaller-Sadovsky, Camilo Hurtado-Parrado, Daniela Cardillo, Lucia G. Medina, Susan G. Friedman

**Affiliations:** 1 School of Biological Sciences, Queen’s University Belfast, Belfast BT95AJ, UK; 2 Department of Psychology, Troy University, Troy, AL 36082, USA; hhurtadoparrado@troy.edu; 3 Faculty of Psychology, Fundación Universitaria Konrad Lorenz, Bogota 110221, Colombia; ligelu25@gmail.com; 4 Green Dogs, Via Dante Alighieri 7, 23814 Cremeno, Italy; danielacardillovspdt@gmail.com; 5 Department of Psychology, Utah State University, Logan, UT 84322, USA; sgfriedman@icloud.com

**Keywords:** conditioned reinforcement, clicker training, dogs, horses, cats, meta-analysis, animal training, behavioral interventions

## Abstract

**Simple Summary:**

Conditioned reinforcement, for example, clicker training, has become increasingly popular in recent decades. Hence, questions about the effectiveness of the conditioned reinforcer have become prominent in the animal training arena. This article summarizes the scientific literature on conditioned reinforcement in applied animal training settings (e.g., homes). It was found that dogs and horses were the most frequently studied animals. Clickers and food were the most often used training stimuli. Effect size analysis found a medium effect of clicker training. The literature reviewed here shows that conditioned reinforcement is an effective approach to change animal behavior; however, sizable information potentially related to its effectiveness was not clearly reported in the studies (e.g., food preferences). Although this review fills in a gap in the literature, it also points to the need for more research to further the understanding of conditioned reinforcement phenomena.

**Abstract:**

A conditioned reinforcer is a stimulus that acquired its effectiveness to increase and maintain a target behavior on the basis of the individual’s history—e.g., pairings with other reinforcers. This systematic review synthesized findings on conditioned reinforcement in the applied animal training field. Thirty-four studies were included in the review and six studies were eligible for a meta-analysis on the effectiveness of behavioral interventions that implemented conditioned reinforcement (e.g., clicks, spoken word, or whistles paired with food). The majority of studies investigated conditioned reinforcement with dogs (47%, n = 16) and horses (30%, n = 10) implementing click–food pairings. All other species (cats, cattle, fish, goats, and monkeys) were equally distributed across types of conditioned (e.g., clicker or spoken word) and unconditioned reinforcers (e.g., food, water, or tactile). A meta-analysis on the effectiveness of conditioned reinforcement in behavioral interventions found a medium summary effect size (Tau-U 0.77; CI_95%_ = [0.53, 0.89]), when comparing baseline, where no training was done, and treatment levels. Moderators of conditioned reinforcement effectiveness were species (e.g., horses) and research design (e.g., multiple-baseline designs). The small number of intervention-focused studies available limits the present findings and highlights the need for more systematic research into the effectiveness of conditioned reinforcement across species.

## 1. Introduction

Humans have been training animals for different purposes for at least 10,000 years [1]. Animal training has traditionally been considered an artisanal skill, mostly encompassing a mix of evidence-based practice (i.e., an intervention or treatment that has been shown to be effective through high-quality and substantial scientific research [2]), personal experience, history, and superstition [3]. Various species, such as domesticated animals (e.g., dogs), insects, fish, and marine mammals are trained across a variety of environments (e.g., homes, shelters, stables, zoos [4,5,6,7,8,9]), applying various training approaches and techniques (e.g., based on positive or negative reinforcement [10]; for a review of training methods [11]). One training approach that has been shown to be applied across species and environments is conditioned positive reinforcement [12].

Historically, conditioned reinforcement builds on the notions of “conditioned reflexes”, also Type S or Type R (i.e., stimulus–stimulus pairing or response–stimulus, respectively), which were first studied and reported by Ivan Pavlov [13] and later reinterpreted by B.F. Skinner as respondent conditioning [14]. Establishing a conditioned reinforcer (e.g., a click, whistle, or spoken word immediately followed by an established reinforcer) is similar to developing a conditioned stimulus in respondent conditioning [15]. Skinner [14] described a procedure in which rats were exposed to a clicking sound paired with food. Later the rats were taught to press a lever by making the sound of the click alone contingent on the lever pressing response. However, because food was no longer delivered, the click was also a respondent extinction trial (i.e., the decline of the eliciting/evocative effect of a conditioned stimulus/reinforcer resulting from presentation of the conditioned stimulus/reinforcer without the unconditioned or well-established conditioned stimulus) [15]. Several years later, the notion of conditioned reinforcement was first formally introduced in the popular press by Skinner [16] (p. 26) in an article published in the *Scientific American* journal, and in an interview of B. F. Skinner featured in the *LOOK* magazine [17]. Skinner [16] (p. 26) described the conditioned reinforcer in this way:

“The best way to reinforce the behavior with the necessary speed is to use a ‘conditioned’ reinforcer. This is a signal which the animal is conditioned to associate with food. The animal is always given food immediately after the signal, and the signal itself then becomes the reinforcer… As soon as the dog moves, sound the cricket and give food.”

Contemporarily, conditioned reinforcement has seen an increase in popularity as a training method since its introduction to a wider animal training audience during the early 1990s—“clicker training” [18,19]. Given its popularity, questions about the effectiveness and efficiency of conditioned reinforcement have become some of the focal points of research in the animal training arena (e.g., [9,20,21,22,23,24,25,26,27,28,29,30]). Generally, studies have either investigated the effectiveness of conditioned reinforcement by teaching animals arbitrarily selected responses (e.g., sliding a lid to open a box, touching the top end of a stick or touching a cone, or spins and bows [20,24,25,26,27,28,29]) or used conditioned reinforcement to teach an alternative behavior during a behavior change program or teaching new skills (e.g., touching a target, slipping into a head halter, or releasing a toy on cue [4,8,22,23]). Different methodologies are evident in this literature. Studies focused on teaching alternative behaviors during a behavior change program (i.e., behavioral interventions) or teaching new skills have generally used clicks consistently followed by food, and each animal serves as its own control (i.e., single-case research methods [31]); in addition, subjects in these studies are often exposed to different experimental conditions (e.g., target training and generalization [4,8,9,22,30,31,32]).

Conversely, research that has investigated the effectiveness of conditioned reinforcement via establishing arbitrary responses has mainly implemented group designs. Animals in the experimental groups have been exclusively exposed to pairings of the target stimulus to be conditioned (e.g., beep, clicker, or spoken word) followed by food, while animals in the comparison groups are trained with food only or conditioned reinforcement only (i.e., no pairings between the target stimulus and food are scheduled [28]). Some of these studies have also investigated resistance to extinction [24,25,26,28,33,34], which has been described in the applied and basic research literature as a procedure to demonstrate the relative effectiveness of conditioned reinforcers (i.e., extinction test [35,36,37]). In contrast, few studies have investigated the efficiency of clicker training by comparing a continuous pairing of click and food versus an intermittent pairing (for a discussion of these points see [38,39]) or click versus spoken word plus food [27,29].

A survey of the literature concerned with the application and effectiveness of conditioned reinforcement showed only two partially related literature reviews, both focused on the possible functions of the clicker (e.g., *bridging, conditioned reinforcement,* or *marking* functions [40,41]). The primary focus of this review is not determining the functional account of clicker training but rather systematically assessing the efficacy of the training procedures described as clicker training. These earlier reports lack a comprehensive systematic review and quantitative analysis (i.e., meta-analysis) of existing applied animal training studies on the application and effectiveness of the clicker. Accordingly, the purpose of the current paper was to (a) update and expand partial earlier reviews on conditioned reinforcement relevant to applied animal training (e.g., systematic searches across a wide range of databases and repositories), (b) synthesize experimental data on conditioned reinforcement across applied animal behavior settings (e.g., homes, training facilities, or stables), (c) quantitatively analyze the effectiveness of conditioned reinforcement when implemented as part of interventions for undesired behavior (e.g., to teach an alternative behavior or new skills to animals). To this end, nonoverlap effect size calculations (Tau-U index) were conducted in the meta-analysis, since single-case research methods (SCRM) were exclusively implemented in the relevant literature. Based on the findings of the review and meta-analysis, potentially promising directions for future research are suggested.

## 2. Methods

This systematic and quantitative review followed the recommendations of Petticrew and Roberts [42] and complied with the Preferred Reporting Items for Systematic Reviews and Meta-Analyses (PRISMA) guidelines [43,44]. A list of keywords for the systematic literature search were acquired by the “Pearl Harvesting Methodological Framework” (PHMF [45]). PHMF has been proven effective in locating the most relevant, inclusive keywords by creating a set of terms referred to as a synonym ring [45,46,47]. This strategy entails the following steps: (a) choosing a representative sample of articles; (b) extracting the relevant search keywords; (c) refining the list of search keywords; and (d) validating the essential search keywords [45,47].

A convenience sample consisting of 10 sources, including book chapters, systematic reviews, and research articles, was used for the keywords’ selection process [12,40,41,48]. The following keywords resulted from applying the PHMF and were used in combination with Boolean operators (where enabled) for database searches:

“clicker training” or “secondary reinforcers” or “secondary reinforcement” or “conditioned reinforcers” or “conditioned reinforcement” or “bridging stimulus” or “event marker” and “dogs” or “cats” or “parrots” or “cows” or “pigs” or “goats” or “horses” or “fish” or “sheep” or “primates” or “pinnipeds” or “cetaceans” and not “humans”

A wildcard, the asterisk (“*”), was used for searching databases and repositories that had such functions enabled.

### 2.1. Literature Search and Study Selection Process

**Figure 1:**
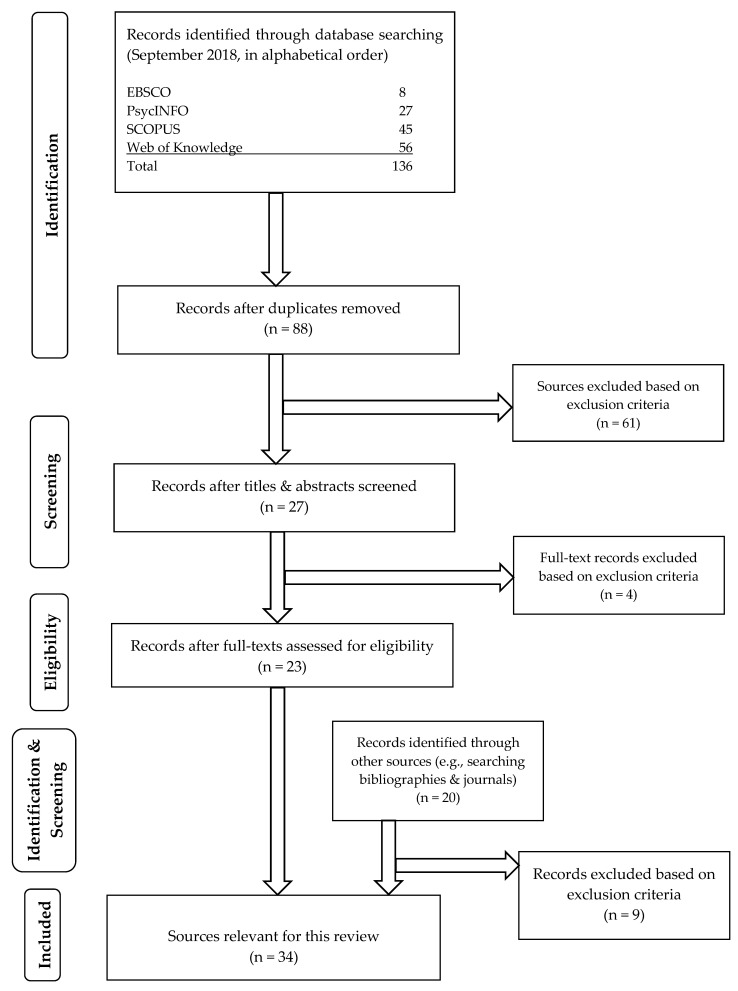
Flow diagram of the separate stages during the selection process for identification of studies eligible for further analysis (adapted after PRISMA guidelines [43,44]).

Studies were located by systematically searching the following databases: SCOPUS, PsychINFO, Web of Knowledge, and EBSCO Open Dissertations. The latter repository was included to reduce the risk of biases in the dataset (e.g., publication bias, which refers to the issue of studies reporting statistically significant results being more likely to be published than studies with less favorable outcomes) [42]. A systematic review process was used to ensure consistency and transparency [43]. Study identification, screening, and eligibility evaluation were conducted using the PRISMA guidelines (see Figure 1) [44]. Methodological restrictions (e.g., between group designs only) and publication date limitations were not implemented. The resulting list of potential studies was screened against exclusion and inclusion criteria. First, the studies’ titles were scanned based on the clearly outlined criteria. If the titles appeared relevant to the proposed review, the abstracts of the studies were read. Second, the full texts of the remaining studies were scrutinized according to the same inclusion criteria to select for eligible papers. Studies were included, if they (a) were original research, including theses and dissertations; (b) were conducted in applied settings, e.g., domestic homes, shelters, stables, zoo enclosures, training facilities, or similar; (c) involved conditioned reinforcement (e.g., auditory, tactile, visual) as the primary independent variable; and (d) used concurrent or non-concurrent observation of animal behavior change (i.e., direct measurement of the effect of the independent variables on dependent variables).

Studies that successfully passed each phase of the screening process were included in the review and subsequent meta-analysis. Additionally, the bibliographies of eligible records and two previous reviews [40,41] were examined for studies not retrieved by database and repository searches. Finally, two journals that previously published studies on applied implementation of conditioned reinforcement (*Applied Animal Behaviour Science* and *Journal of Applied Behavior Analysis*) were hand-searched for relevant articles that met inclusion criteria. To summarize, the implemented search strategies and selection processes were conducted from September 2018 through March 2019 and yielded a total of 34 eligible studies.

To ensure the accuracy of relevance decisions throughout the screening process, a second examiner (DC) unaware of the aims of the review independently examined 35 out of 136 records’ abstracts (i.e., >25%) against exclusion and inclusion criteria. Studies appraised by the second examiner (DC) were randomly selected using the Microsoft^®^ Excel^TM^ application “random function”. Each examiner’s (NPS and DC) agreements and disagreements of the selected records were compared, and an inter-examiner agreement (IEA) score was calculated by number of agreements divided by number of agreements plus number of disagreements multiplied by 100. IEA computation resulted in a 91% agreement score across both examiners. Any disagreements were resolved by discussion.

### 2.2. Assessment of Studies and Variable Coding

For coding the variables of interest, a specifically designed Microsoft^®^ Excel^TM^ matrix was employed (see copy available at Open Science Framework (OSF), doi:10.17605/OSF.IO/V5MHF). All remaining 34 studies were coded on the following criteria: (a) bibliographic information (e.g., authors, publication date and type, and country); (b) sample size; (c) description of selection of sample (e.g., species of subjects); (d) information on procedures, such as if pairing sessions were conducted, if interstimulus intervals were measured, or details on the type and delivery of the unconditioned reinforcer (e.g., food delivered in a container; see Table 1 for definitions); (e) description of subjects’ target behavior (i.e., dependent variables, e.g., touching a cone with their muzzle); (f) description of the treatment (i.e., independent variables, e.g., click and food). Whenever more than one independent variable was implemented, additional rows were introduced to account for the different comparisons (see Table 2) [49]; (g) type of research design (e.g., between-group designs and single-case research methods); and (h) effect size indices (e.g., Tau-U for single-case research methods). If effect sizes were not given in the original studies, they were calculated by using data provided in the records.

To maintain consistency in coding, a second examiner (DC) independently coded 25% of all eligible articles (i.e., 9 out of 34). These studies were again randomly selected by employing the random function, a Microsoft^®^ Excel^TM^ application. The inter-coder agreement score (ICA) was determined by applying the item-by-item calculation method (i.e., number of agreements divided by number of agreements plus number of disagreements multiplied by 100). These calculations were done for each of the 38 items (e.g., “setting, training location” or “independent” and “dependent variables”) and yielded an ICA of 92% across both coders. Any disagreements were resolved by discussion.

The overall study quality was assessed by a research assistant (DC) using a 14-item checklist adapted after Logan, Hickman, Harris, and Heriza [52]. To ensure consistency during the quality assessment process, another trained research assistant (LGM) assessed 30% of all eligible studies (i.e., 11 out of 34). The same random study selection approach and item-by-item computation method as before were used and yielded an agreement score of 97% across both reviewers. As previously, any disagreements were resolved by discussion.

### 2.3. Data Extraction and Meta-Statistics

Six out of seven SCRM studies reported suitable data for further analysis [9]. Given there were three or more studies allowing for comparison, they were combined according to research design (e.g., multiple-baseline across-subjects) and whether the effectiveness of conditioned reinforcement was investigated or not (e.g., conditioned reinforcement was part of the intervention for an undesired behavior or to teach a new skill).

Data were extracted from graphs reported in the SCRM studies using the free DigitizeIt Version 2.4.0 [53] online software. A PNG image of each graph was pasted into the software and the coordinates, as well as data points, were plotted. The resulting digitized data of baseline and interventions for each AB contrast were exported to a Microsoft^®^ Excel^TM^ file.

The *Tau-U* effect size index was computed for each study and for potential moderator variables. This index describes the “percent nonoverlapping data minus the percent of overlapping data” [54] (p. 285). It was selected as the index for the current analysis because it has been found to be consistent with visual analysis of SCRM data, has the ability to control for an undesired baseline trend (i.e., trend in the direction of the intervention or confounding direction), is robust enough for small data sets, and was shown to have greater statistical power and precision compared to other nonoverlap effect size indices [55,56]. Tau-U is interpretable as a continuous index of improvement and the published benchmarks should be considered relative to participants’ needs, intervention comparisons, and settings: 0 to 0.62 = small effect; 0.63 to 0.92 = medium effect; 0.93 to 1.00 = large effect [56,57]. The Tau-U indices were computed by entering baseline and intervention data into a free online Tau-U calculator [58]. An effect size value was obtained for each AB contrast (i.e., baseline versus intervention). These experiment-level effect sizes were then combined into one omnibus effect size per study by applying an inverse weighting computation that puts more weight on studies with more data points and stability [57].

During the next step, effect sizes were entered into the Comprehensive Meta-Analysis^®^ software program (Version 3.3; [59]) to generate a summary effect size which represents all included studies. The summary effect size reflects a weighting computation that assigns relevance based on within-study and between-study variance [57].

Although neither a random nor fixed effects model is considered perfectly suitable for SCRM data, the assumed across-studies differences (e.g., subjects, outcome measures, procedures, and settings) made a random effects model the most appropriate [57]. In other words, the variance between studies was hypothesized to be due to systematic differences rather than sampling error, hence, implementation of a random effects model seemed to be appropriate for the current analysis [49,60].

Additionally, the application of a random effects model additionally allowed for assessment of the presence of moderator variables (i.e., covariates which may have an impact on the effect size; [60,61]). Moderator analyses were conducted by entering the Tau-U indices and respective standard errors for each AB contrast into the meta-statistic software as done previously in [59], yielding an effect size and additional statistical measures (e.g., standard error [SE] and *p*-values) for each potential moderator.

#### Heterogeneity Assessment

Heterogeneity or between-study variation is defined as the degree to which each study’s effect size varies within the dispersion (range) of effect sizes [61]. In other words, the variation described by heterogeneity cannot be accounted for by sampling error [62,63]. The *Q*- and *I^2^*-statistics were used to assess heterogeneity in this analysis [64]. Essentially, the *Q*-statistic is a test that assesses the null hypothesis stating that studies share a common effect size, with alpha typically set at 0.05, and *p*-values less than alpha leading to rejection of the null (i.e., studies do not share a common effect size [64]). The *I^2^* index was calculated and can be described as the percentage of variance between studies [65]. A general guideline for the interpretation of the *I^2^* index was put forward by Higgins and Green [62], stating that *I^2^* > 75% should be considered substantial heterogeneity between study effects.

## 3. Results

The characteristics of all studies (n = 34) included in the systematic review are displayed in Table 2. The SCRM studies that were eligible for further meta-statistical analysis are highlighted in bold.

### 3.1. Systematic Review

#### 3.1.1. Search Results and Study Characteristics

**Figure 2:**
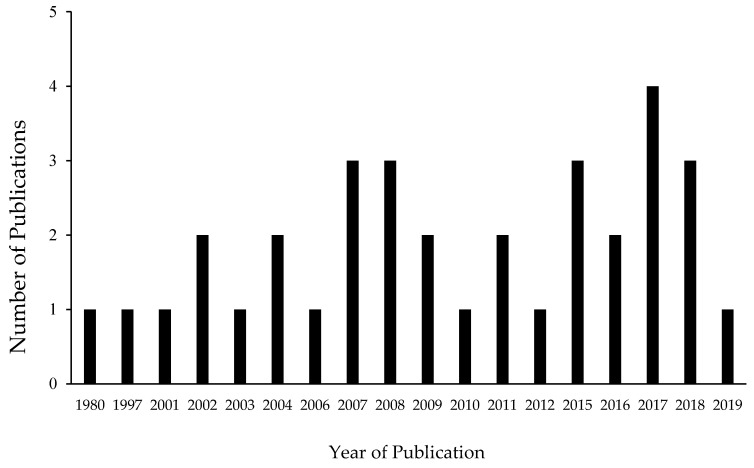
Numbers of publications per year across all studies included in the review.

The searches yielded 136 sources from four databases and repositories, such as PsycInfo and SCOPUS. After completion of the selection process (Figure 1), 34 sources were found relevant for the current systematic review. Out of these 34 eligible studies published between 1980 and 2019 (Figure 2), the majority of studies (29%, n = 10) were published in the *Applied Animal Behaviour Science* journal using some type of group comparison design (“group design”; e.g., nonequivalent controlled or randomized controlled). The second largest category was theses (21%, n = 7), featuring mainly studies using SCRMs (12%, n = 4).

Overall, the majority of studies (59%, n = 20) used some type of group design, seven studies (21%) used SCRMs (e.g., multiple-baseline across-subjects design or reversal design), while another seven studies (21%) were categorized as case studies due to insufficient reporting of data and generally being more narrative in nature (see copy of respective table available at Open Science Framework (OSF), doi:10.17605/OSF.IO/V5MHF).

#### 3.1.2. Main Findings of the Systematic Review

As shown in Table 3, almost half of all included studies (47%, n = 16) reported investigating the effects of conditioned reinforcement in dogs. Thirty-two percent (n = 11) of the studies with dogs used clickers as conditioned reinforcers and *food (delivered by hand)* as the unconditioned reinforcer. One study each (3%, n = 1) implemented either a *spoken word* and food delivered by hand or a whistle with *food (presented in proximity)* to the trainer. Horses followed dogs in number of studies (30%, n = 10). The majority of research with horses implemented pairings of clickers with food (24%, n = 8), with five studies (15%) delivering the food by hand and two studies (6%) presenting the food in a container. All other species (cats, cattle, fish, goats, and monkeys) were equally distributed across *types of conditioned reinforcers* with one study each (3%, n = 1).

##### Unconditioned Reinforcers (S^R+^) and Preference Assessments

Only two out of 34 studies (6%) conducted *preference assessments* (i.e., selection of alternative reinforcement options, where one alternative is selected more frequently leading to the identification of preference for a certain activity, food, or person [15,79]). One study used food delivered by hand with dogs [20], while the second study with foals used scratching as the unconditioned reinforcer [50]. To summarize, out of 30 studies using food as an unconditioned reinforcer with any form of delivery (e.g., by hand or in a container), the vast majority of studies (90%, n = 27) did not perform a preference assessment to identify their learner’s preferred reinforcers. Table 4 provides detailed information.

##### Schedules of Reinforcement

Learner performance depends on the schedules of reinforcement implemented; similar schedule effects have been found across organisms, types of responses, and a variety of reinforcers [15]. Two broad categories of schedules of reinforcement, namely continuous and intermittent, were coded in the identified studies. With continuous reinforcement, each response produces reinforcement (e.g., every response produces a click followed by food [27]). With an intermittent schedule of reinforcement, some responses are reinforced, while reinforcement is withheld on some occasions (i.e., conditioned or unconditioned reinforcers [80]). For instance, on a variable ratio 2 schedule, on average, every two responses produce an conditioned reinforcer followed by food [15,26]. The current review found that 85% (n = 29) of the studies delivered the unconditioned reinforcers on a continuous schedule of reinforcement, irrespective of whether a conditioned reinforcer was used, or only the unconditioned reinforcer was presented (e.g., click followed by food or food only). Overall, 24% (n = 8) of studies reported the implementation of intermittent schedules (e.g., a variable ratio schedule was implemented [26]), and 12% (n = 4) of studies did not clearly state the schedule used (see copy of respective figure available at Open Science Framework (OSF), doi:10.17605/OSF.IO/V5MHF).

##### Interstimulus-Intervals (ISI), Delays between Responses and Conditioned Reinforcers (R − S^r+^), and Number of Pairings

The close temporal proximity between two events, i.e., contiguity, has been found to be important for stimulus–stimulus learning, and therefore has been deemed necessary for establishing a conditioned reinforcer [15,80]. Based on these methodological aspects of conditioned reinforcers, three different time intervals, as well as information on pairing sessions, were coded: (a) number of conditioned stimulus (CS) and unconditioned stimulus (US) pairings (e.g., ≤20 pairings per day), (b) *interstimulus interval* (ISI) between CS and US during pairing sessions (i.e., subjects were only exposed to the stimuli, without reinforcement of a target response; Table 5), as well as during training (i.e., when the pairings of S^r+^ and S^R+^ occurred naturally as part of the reinforcement of a target response), and (c) delay between target response and S^r+^. Table 5, Table 6 and Table 7 report these findings in detail. Surprisingly, the majority of studies (44%, n = 15) did not clearly state whether pairings have been scheduled and the amount or the length of intervals if pairing was applied. Almost a third of the studies (29%, n = 10) reported that no explicit pairing procedure was conducted, followed by a number of studies that implemented a maximum of 20 pairings per day (27%, n = 9; Table 6).

With regard to the delay of the conditioned reinforcer (i.e., delay between the target response and the presentation of the conditioned reinforcer; R → S^r+^), a third of the studies used five different descriptions (32%, n = 11), such as “immediately after the response occurred” or “shortly after”. The majority of studies (68%, n = 23), however, did not clearly state the delay between the target response and conditioned reinforcer presentation (Table 6).

Similarly, nonspecific statements have been found for reporting of the interval that elapsed between the conditioned reinforcement presentation (e.g., click; S^r+^) and the delivery of the unconditioned reinforcers (e.g., food, water, or scratching; S^R+^). Although 15% (n = 5) of studies reported the delivery of the unconditioned reinforcer “immediately” after the conditioned reinforcer, more than half of all studies (59%, n = 20) did not clearly state the specific time (e.g., seconds) that elapsed between these two stimulus events (Table 7). To summarize, these findings lacked specific information about the measurement of interstimulus and response–reinforcer delay intervals across the applied animal training literature.

##### Positioning of the Experimenters and Location of the Unconditioned Reinforcers (S^R+^)

Based on the input received from renowned experts in the field (e.g., professional animal trainers and senior researchers), and some anecdotal observations reported in the literature [81], the positioning of the experimenter or trainer and the location of respective unconditioned reinforcers were coded (e.g., experimenter or trainer was located outside the view of the animal with food in close proximity to them but the food container not attached [23]). Table 8 displays this information by species. Consistent with the overall tendency to omit reporting of some features of the experimental set up and procedures identified so far, more than half of the studies (53%, n = 18) did not provide information on the positioning of experimenters and respective unconditioned reinforcers. Among the remaining studies, the majority described the experimenter being positioned in front of the learner (36%, n = 12), of which 24% (n = 8) were canine studies reporting reinforcers were delivered in front of the dog from a container attached to the body of the experimenter (e.g., treat pouch [76]). A less frequent variation of this arrangement (9%, n = 3) that was described in studies with fish, horses, and monkeys was the experimenter positioned in front of the learner and the reinforcer being delivered in proximity.

#### 3.1.3. Meta-Statistical Results

For the current meta-analytic review, only SCRM designs were considered. This decision was reached because we were mainly interested in clicker training’s effectiveness within the behavioral intervention realm. Six out of seven studies reported suitable data for effect size calculations (included studies are highlighted in bold in Table 2). Three different species (dogs, horses, and goats), totaling 23 subjects across six studies, and encompassing 52 AB phase contrasts, were found. The majority of studies (66.6%, n = 4) used multiple-baseline designs (MBL), whereas one study applied either a reversal (RVD) or a changing criterion design (CCD; 33.3%). Small, medium, and large effects were equally distributed across all six studies, yielding two studies for each classification (33.3% each). Table 9 displays the studies’ characteristics and individual Tau-U effect sizes.

##### Effects of Clicker Training

Overall, Tau-U effect sizes for the use of clicker training to change an undesired behavior (e.g., separation-related responses in horses [23]) or to teach animals new responses (e.g., wearing head halters in goats [4]) ranged from 0.48 to 0.98, with a mean or summary effect size of Tau 0.77 (CI_95%_ = [0.53, 0.89]) when compared to baseline levels. In other words, clicker training yielded a medium effect, regardless of animal species and type of target behavior (e.g., nose target, bowing, delivering object to hand, or slipping head into halter).

While five studies applied clicker training for teaching new skills to their animals on a continuous schedule only (i.e., each correct response resulted in click then food), Wennmacher [27] investigated the effect of click and continuous pairing of food versus click and intermittent presentation of food (i.e., each correct response resulted in a click but food was given only after every second click). Although small (Tau-U 0.48; CI_95%_ = [0.33, 0.61]), the effect size indicates that continuous pairing with food was slightly more effective in teaching new responses to dogs than delivering food intermittently.

**Figure 3:**
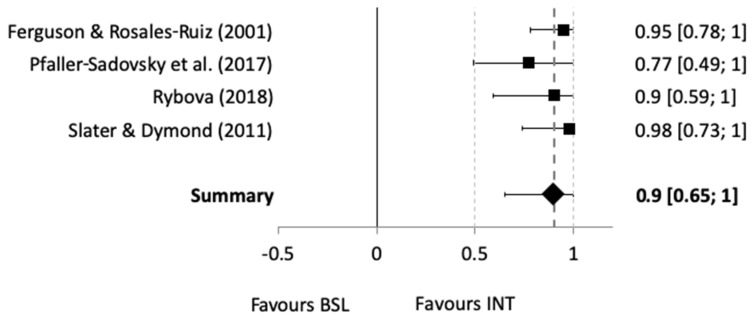
Forest plot featuring the studies MBL studies with respective Tau-U effect sizes. The diamond-shaped data point at the bottom of the plot represents the mean or summary effect size across all four studies. Note: BSL = baseline; INT = intervention.

The majority of studies implemented MBLs, hence a separate comparison of these studies could be conducted, and a forest plot was created (Figure 3). These four studies were also those that implemented clicker training as part of their intervention to increase alternative behavior (e.g., trailer loading with horses or delivering a preferred item to hand with dogs). Overall, studies using MBLs yielded medium to large effects, with a summary effect size of Tau-U 0.90 (CI_95%_ = [0.65, 1]). Put differently, MBL studies using clicker training as part of their behavior change interventions had a medium effect favoring the intervention.

##### Heterogeneity Assessment

Although the sample size of studies eligible for quantitative analysis was small, an assessment of how dissimilar the included studies are (i.e., heterogeneity assessment) was attempted. The heterogeneity assessment yielded a statistically significant result (*p* = 0.00), therefore the null hypothesis (i.e., all studies share a common effect size) could be rejected. Additional statistical results, namely *Q* = 23.83 and *I*^2^ = 79.02%, suggest that the studies are heterogeneous and that almost all of the variance may be explained by differences in study characteristics.

##### Moderator Analyses

Ten variables related to various study characteristics (e.g., type of conditioned reinforcer, amount of pairing sessions, or species) were examined for their potential impact on the effectiveness of clicker training. However, four variables could not be further analyzed due to insufficient data (e.g., type of conditioned and unconditioned reinforcers and schedule of reinforcement) or too small sample size (e.g., contiguity of conditioned and unconditioned reinforcers and prompts used). For the remaining six variables, moderator analyses were conducted by computing meta-regressions using the Comprehensive Meta-Analysis^®^ software [59].

Two of the potential moderators yielded statistically significant results (*p* < 0.05), namely *learner species* and *study design* (Table 10). These variables seemed to have influenced the effectiveness of clicker training in the current set of studies. The covariate *horses* yielded larger effect sizes than goats or dogs (0.943, 0.574, and 0.573, respectively). Studies implementing an MBL design had higher effects than studies using changing criterion or reversal designs (0.92, 0.34, and 0.44, respectively).

To summarize, the current analysis showed that clicker training resulted in mainly medium effects (range, 0.63 to 0.92) across a range of species (i.e., horses, dogs, and goats) and target responses taught (e.g., nose touches, spin and bow, and delivering a preferred item to hand). However, these results should be interpreted cautiously due to the very limited amount of studies that were available for inclusion in this quantitative analysis (n = 6).

## 4. Discussion

A total of 34 studies were found eligible after the selection process was completed (Figure 1). The majority of studies were undertaken with dogs and horses, using the sound of clickers paired with food. Studies that reported the use of conditioned reinforcement during behavioral intervention were chosen for further meta-statistical analyses. Six studies reported sufficient detail to be eligible for further analysis. These studies paired clickers with food, which yielded a medium effect when compared to baseline measures (summary effect of Tau-U 0.90; CI_95%_ = [0.65, 1]; range from 0.63 to 0.92). These effects were found regardless of animal species (dogs or horses), type of target behavior taught during the behavior change intervention (e.g., nose target, delivering object to hand, or slipping head into a halter), and design used (e.g., MBL across-subjects design).

### 4.1. Systematic Review

This review found that the majority of eligible studies used some type of group design, whereas only a small number of studies implemented SCRMs. SCRMs are closely associated to the behavior analytic approach [82]. Thus, the small number of SCRM studies identified here further supports the notion that there is an overall lack of behavior-analytic research in the applied animal behavior change arena [83], including the application of conditioned reinforcement across applied settings and species. Two earlier reviews on the use of conditioned reinforcement in the animal behavior realm reached similar conclusions (e.g., clicker training [40,41]). This trend in the literature is surprising, as several authors have pointed out that reinforcement-based animal training, including conditioned reinforcement, is firmly rooted in behavior analysis [3,18,84,85], and has been shown to be successful in improving a wide range of socially relevant behaviors across settings and species (e.g., fear-related behavior in dogs and sheep [86,87,88]). Further investigation seems needed to explore and, hopefully improve, conceptual and methodological cohesiveness in the implementation of procedures with a behavior-analytic tradition in applied animal contexts [89,90].

The distribution of the identified studies across the different sources showed that the majority were either published in two clinical/applied journals (*Applied Animal Behaviour Science* and *Journal of Veterinary Behavior: Clinical Applications and Research*) or were available as theses/dissertations. These findings suggest that most research related to conditioned reinforcement has had clinical/applied implications. Further, an important number of studies have not been published as journal articles. The latter finding seems to be aligned with previous reports across other disciplines about the proportion of these clinical/applied-oriented dissertations that do not get published in indexed journals [91]. An explanation of this situation goes beyond the scope of this review, but it has been noted that applied dissertations/theses often do not get published because the authors do not have expectations of careers in academia, where publications are valued [91,92].

Almost half of the identified studies were conducted with dogs, and the second most frequently studied subjects were horses; in both cases, clickers and food were the most frequently paired stimuli. The remaining studies focused on a wide range of species, including cats, goats, fish, cattle, and monkeys, and additional conditioned stimuli included beeps, whistle and buzzing sounds, and spoken words. Although the number of studies per these species and stimuli was limited, evidence shows promise of a wide generality of conditioned reinforcement phenomena and procedures across species, responses, and settings [30,93,94,95,96].

Regarding the type of unconditioned reinforcer (S^R+^) applied, the vast majority of studies used food. Out of these studies, only one study reported the implementation of some form of reinforcer preference assessment, though it was not systematic in nature (i.e., a variety of food rewards was presented to the owners and/or the dogs to choose from). This lack of preference assessment in the identified literature is an unexpected finding, since a growing body of literature has already highlighted the value of conducting preference assessments in animal behavior interventions across the settings, species, and stimuli examined (e.g., food or toys [79,97,98,99,100]). Overall, research shows that the delivery of a pre-established preferred stimulus as a reinforcer may improve the intervention outcome relative to the use of arbitrarily selected items with a presumed reinforcing function (e.g., assumed by the animals’ caregivers [79,99,101]).

Our findings have highlighted the lack of measurement and/or reporting of different temporal parameters, such as the ISI between the conditioned and unconditioned stimuli [80] and delay between the target response and reinforcement [102]. This is important given the many formal and informal animal training and learning resources [3,103,104,105,106,107] that highlight the importance of contiguity between events on the effectiveness of conditioned reinforcement in practice [15,80].

In the context of respondent (Pavlovian or classical) conditioning, contiguity is defined as the close temporal proximity between the conditioned stimulus and the unconditioned stimulus during the conditioning procedure [15]. The use of this respondent procedure to “charge up the clicker” prior to the operant training of target responses is often mentioned in the animal training literature as a foundational part of clicker training with an inexperienced animal [106,107]. The extent to which the pairing is needed is itself an empirical question, not addressed in the literature identified for the present review. There does not seem to be a consensus regarding the number of pairings needed to establish the previously neutral stimulus as a conditioned stimulus [16,36,37,108,109]. Similar to Feng et al. [108], this review also found considerable variation in the number of pairings reported. For example, almost a quarter of the studies implemented a maximum of 20 pairings per day [28], fewer studies reported on up to 60 pairings per day [66]. Almost a third of the studies, however, did not report implementing extra pairings prior to the onset of the actual operant training phase [76]. This is in line with the findings of Feng et al. [108,110], who also found large variations in numbers of pairings reported across animal trainers working in the industry and applied animal behavior research papers. Taken together, these findings highlight the need for applied research that systematically manipulates, across species and settings, the number of pairings as an independent variable.

Lastly, two procedural aspects with a wide range of variation and lack of information across the relevant studies were the position of the experimenter/trainer, and the location of the unconditioned stimulus (S^R+^). More than half of the studies did not provide information on these two aspects. Among the remaining studies that did provide some information, the majority were canine studies that described the experimenter being positioned in front of the learner and reinforcers delivered in front of the dog from a container attached to the body of the experimenter. Similar to the temporal aspects of the presentation of the conditioned and unconditioned stimuli, the spatial relation between the conditioned and unconditioned stimuli seems to affect the establishment of the respondent conditioning [111]. For example, depending on the distance between the conditioned and unconditioned stimuli, pigeons may develop conditioned responses that include approaching the conditioned stimulus (sign tracking), approaching the site where the US is presented (goal tracking), or a combination of both sign and goal tracking [112]. The fact that several studies did not report information on the location of the experimenter and/or the place where the unconditioned stimulus was delivered suggests future research could explore how these factors affect the effectiveness of conditioned reinforcement procedures, including the emergence of unexpected responses (goal or sign tracking) that perhaps relate to spatial configurations of the conditioned and unconditioned stimulus.

### 4.2. Meta-Analysis

The present meta-analysis aimed to quantify the effectiveness of clicker training as part of interventions to change animal behavior in applied settings, which was not reported in the literature previously. All the studies of relevance to this aim implemented SCRMs, which most likely could be the result of these methods being best suited to experimentally assess behavior change at the individual level (i.e., the causal relations between one or more IVs and DVs [84,113]).

Overall, the Tau-U summary effect sizes demonstrated that the interventions using clicker training were effective in changing the learners’ behavior, resulting in small to large effect sizes (Tau-U range, 0.48–0.98, CI_95%_ = [0.3, 1]), irrespective of their species and setting (e.g., dogs, goats, and horses in homes, enclosures, and stables, respectively). It has to be noted that the numerical benchmarks (e.g., 0 to 0.62 = small effect; 0.63 to 0.92 = medium effect; 0.93 to 1.00 = large effect [50,51]) should be interpreted contextually and with caution as small effects can result in important improvements on the learners’ behavior and welfare [11,56].

In agreement with published behavior-analytic research in the human behavior change arena [114,115,116,117], MBL across-subjects designs were the most frequently used type of SCRM, which yielded a medium summary effect size (Tau-U 0.90, CI_95%_ = [0.65, 1]). A moderator analysis for MBL across-subjects designs (meta-regression; Table 10) found that this type of MBL design had an impact on the effectiveness of the interventions across all species. One factor contributing to this finding may be that the majority of analyzed studies used such designs (n = 4), while only one study implemented a changing criterion design, and another study used a reversal design. Although it is well established that MBL designs have several advantages compared to other SCRM designs, such as withdrawal of the intervention or reversal to baseline is not required [84,113], the results have to be interpreted carefully due to the overall small number of studies included in the analysis.

The meta-regressions computed for the analysis of potential effect moderators yielded two statistically significant results; namely, *learner species* and *study design* (Table 10). While the latter has been addressed in the MBL across-subjects design section above, the former is discussed in the remainder of this section. The variable *learner species* had moderating capabilities for the effectiveness of clicker training, yielding a larger effect for horses than for dogs and goats. Although the finding that learner species can influence the effectiveness of interventions is in line with an earlier meta-analysis investigating the effectiveness of caregiver training programs for both human and non-human animal learners [114], the current findings should be interpreted carefully. Half of the eligible studies used horses, while two studies used dogs and only one study featured goats. It is very likely that the overall small number of studies and the majority of studies using horses contributed to this outcome.

### 4.3. Limitations

There are several limitations which should be noted. First, dividing the dataset according to research designs was based on (a) the interest of analyzing studies aimed at changing behavior, which happened to use SCRMs, and (b) the lack of commonly accepted effect size indices that could be used to synthesize SCRM and group research [57]. Therefore, only a very small number of studies could be included in the meta-analysis, which makes the interpretation of results, especially the moderator analyses, tentative. Lastly, the application of Tau-U, or other nonparametric effect sizes (e.g., percentage nonoverlapping data; PND [118]), may lead to the loss of individual characteristics of behavior change patterns in SCRM data when averaged, which could make conclusions misleading [57,119].

## 5. Conclusions

Our quantitative review found that the majority of eligible studies were conducted with dogs and horses, and most studies applied clickers and food as conditioned and unconditioned reinforcers. While we identified conditioned reinforcement (i.e., clicker training) as an effective approach to change animal behavior, many of the variables that seem to affect its effectiveness could not be unambiguously retrieved from the eligible studies (e.g., contiguity and contingency). Hence, we identified several potential avenues for future research, such as systematically manipulating (a) time intervals or delays between response and the onset of the conditioned reinforcer or the end of the conditioned reinforcer and the delivery of the unconditioned stimulus, (b) type of conditioned reinforcers other than clickers and spoken words (e.g., whistles or beeps), and (c) types of unconditioned reinforcers or back-up reinforcers presented after the delivery of the conditioned reinforcer (e.g., different food items, play, or tactile interaction based on preference assessments [120,121]). Future research efforts could also focus on the replication of studies across different species (e.g., companion pigs or rodents) and settings (e.g., enclosures or domestic homes). These efforts may also provide a translational approach (i.e., the synthesis between basic and applied research leading to a bidirectional flow of information and influence [122,123]) to further the understanding of fundamental behavioral principles and processes [124].

## Figures and Tables

**Table 1:** Details on the delivery of the S^R+^ and respective definitions as reported in the eligible studies.

Details on S^R+^ Delivery	Definitions
Food by hand	Food was brought to the animals’ muzzles by hand (e.g., dogs, and horses) or was presented by hand in a manner that the animals could reach for the S^R+^ with a forelimb (e.g., rhesus macaques or squirrel monkeys).
Food remotely	Food was delivered through an automated feeder which was remotely controlled by the experimenter.
Food container	Food was made available to the animal in a container (e.g., feedbox for horses or a food bowl for dogs).
Food proximity	The food was consistently delivered in the same place in close proximity to the experimenter (e.g., after the animal performed the task, the experimenter delivered the conditioned reinforcer and placed the food near to him/her on the floor [20]).
Scratching	Scratching (e.g., chest neck or rump) was used as an alternative S^R+^ to food for foals because they were still nursing during the experiment [50].
Water	Water was available as an S^R+^ in a computer-controlled learning device located in a separate compartment of the home pen (stable) of the goats. The animals had 24 h access to the device, but only one goat could enter it at a time [51].
No S^R+^	Only the S^r+^ (conditioned reinforcer) was presented or reinforcement was entirely withheld (i.e., extinction).
Not clearly stated	Type of S^R+^ and mode of delivery were not clearly stated.

**Table 2:** Characteristics of all eligible studies.

Doc ID	Study (Year)	Country	Publication Type	Learner Species	Learner Sex	Learner Age	Type of Conditioned Reinforcer	Setting, Training Location	Type of Study	Learner Target Behavior	Dependent Variables	Independent Variables
1/1	Batt et al. (2008) [66]	Australia	Peer-reviewed paper	Dog (*Canis familiaris*)	>50% male intact	≤4 months	Clicker	Community hall	Group design	Attention, leave it, stand, loose-leash walking	Re-adoption of subsequent puppies	Conditioned Reinforcement and food
1/2	Batt et al. (2008) [66]	Australia	Peer-reviewed paper	Dog (*Canis familiaris*)	>50% male intact	≤4 months	Not applicable	Community hall	Group design	Attention, leave it, stand, loose-leash walking	Re-adoption of subsequent puppies	Control (treatment as usual)
2/1	Blandina (2010) [67]	USA	Bachelor thesis	Dog (*Canis familiaris*)	Not clearly stated	≤6 months	Clicker	Shelter room	Group design	Stay	Duration	Conditioned Reinforcement and food
2/2	Blandina (2010) [67]	USA	Bachelor thesis	Dog (*Canis familiaris*)	Not clearly stated	≤6 months	Spoken word	Shelter room	Group design	Stay	Duration	Conditioned Reinforcement and food
2/3	Blandina (2010) [67]	USA	Bachelor thesis	Dog (*Canis familiaris*)	Not clearly stated	≤6 months	Not applicable	Shelter room	Group design	Stay	Duration	Food only
3/1	Chiandetti et al. (2016) [20]	Italy	Peer-reviewed paper	Dog (*Canis familiaris*)	>50% female intact	1–5 years	Clicker	Home	Group design	Open box by pushing up the lid with nose	Number of attempts over time	Conditioned Reinforcement and food
3/2	Chiandetti et al. (2016) [20]	Italy	Peer-reviewed paper	Dog (*Canis familiaris*)	>50% female intact	1–5 years	Spoken word	Home	Group design	Open box by pushing up the lid with nose	Number of attempts over time	Conditioned Reinforcement and food
3/3	Chiandetti et al. (2016) [20]	Italy	Peer-reviewed paper	Dog (*Canis familiaris*)	>50% female intact	1–5 years	Not applicable	Home	Group design	Open box by pushing up the lid with nose	Number of attempts over time	Food only
4/1	D’Onofrio (2015) [32]	USA	Master’s thesis	Dog (*Canis familiaris*)	50% female	1–9 years	Clicker	Home	Single-case method	Retrieve medicine bag and pick up wallet	Frequency	Conditioned Reinforcement and food
5/1	Feng et al. (2018) [21]	Australia	Peer-reviewed paper	Dog (*Canis familiaris*)	>50% male neutered	1–5 years	Clicker	Home	Group design	Spin, hand target, object target, on your mat	Frequency	Conditioned Reinforcement and food
5/3	Feng et al. (2018) [21]	Australia	Peer-reviewed paper	Dog (*Canis familiaris*)	>50% male neutered	1–5 years	Not applicable	Home	Group design	Spin, hand target, object target, on your mat	Frequency	Control (waiting list)
**6/1**	**Ferguson et al. (2001) [22]**	**USA**	**Peer-reviewed paper**	**Horse (*Equus caballus*)**	**Female intact**	**5–18 years**	**Clicker**	**Outdoor enclosure**	**Single-case method**	**Nose touch to a cloth potholder**	**Latency**	**Conditioned Reinforcement and food**
**6/2**	**Ferguson et al. (2001) [22]**	**USA**	**Peer-reviewed paper**	**Horse (*Equus caballus*)**	**Female intact**	**5–18 years**	**Clicker**	**Outdoor enclosure**	**Single-case method**	**Nose touch to a cloth potholder**	**Parts of horse entering**	**Conditioned Reinforcement and food**
**7/1**	**Fernandez (2003) [4]**	**USA**	**Master’s thesis**	**Goat (*Capra hircus*)**	**Male neutered**	**<12 months**	**Clicker**	**Outdoor enclosure**	**Single-case method**	**Slipping head into halter**	**Shaping steps completed**	**Conditioned Reinforcement and food**
8/1	Fernström et al. (2009) [68]	Sweden	Peer-reviewed paper	Rhesus monkey (*Macaca mulatta*)	Female intact	1–5 years	Clicker	Indoor enclosure	Group design	Target, cooperative behavior, box and injections	Number of sessions to completion	Treatment intensities
9/1	Fjellanger et al. (2002) [69]	Norway	Peer-reviewed paper	Dog (*Canis familiaris*)	>50% male intact	1–5 years	Whistle	Training facility	Case study	Scent discrimination (explosives)	Proportion correct responses	Conditioned Reinforcement and INT food
10/1	Flannery (1997) [70]	USA	Peer-reviewed paper	Horse (*Equus caballus*)	Not clearly stated	12–24 years	Clicker	Stable	Case study	Touching labeled stimulus cards (discrimination task)	Percent correct trials	Conditioned Reinforcement and food
10/2	Flannery (1997) [70]	USA	Peer-reviewed paper	Horse (*Equus caballus*)	Not clearly stated	12–24 years	Clicker	Stable	Case study	Higher-order discrimination task	Percent correct trials	Conditioned Reinforcement and INT food
11/1	Flynn (1980) [71]	USA	Master’s thesis	Horse (*Equus caballus*)	Female intact	>15 years	Clicker	Stable	Group design	Walk around cone, open mouth, stepping diagonally, still for 60 sec, nodding	Count correct responses	Conditioned Reinforcement and INT food
12/1	Fugazza et al. (2015) [72]	Italy	Peer-reviewed paper	Dog (*Canis familiaris*)	Not clearly stated	1–12 years	Clicker	Training facility	Group design	Open a sliding door, jumping in the air	Latency	Conditioned Reinforcement and food
12/2	Fugazza et al. (2015) [72]	Italy	Peer-reviewed paper	Dog (*Canis familiaris*)	Not clearly stated	1–12 years	Not applicable	Training facility	Group design	Open a sliding door, jumping in the air	Latency	Modeling the response
13/1	Gillis et al. (2012) [5]	USA	Peer-reviewed paper	Squirrel monkey (*Saimiri boliviensis*)	Male intact	1–5 years	Clicker	Laboratory	Case study	Target and duration training, glove desensitization, chain and pole, injection training	Time to criterion	Conditioned Reinforcement and food
14/1	Grant et al. (2019) [6]	UK	Peer-reviewed paper	Cat (*Cattus syslvestris*)	>50% male neutered	Not clearly stated	Clicker	Shelter room	Group design	Duration exploratory behavior	Duration	Conditioned Reinforcement and food
15/1	Guerrero-Flores et al. (2017) [73]	Mexico	Peer-reviewed paper	Dog (*Canis familiaris*)	Male intact	1–5 years	Clicker	Laboratory	Case study	Indicate target odor by sitting in front of sample	Frequency	Conditioned Reinforcement and food
16/1	Häderer et al. (2016) [33]	France	Peer-reviewed paper	Fish (*Tripterygion tripteronotum*)	Mixed	Not clearly stated	Clicker	Natural environment/home range	Group design	Target black/white chip with muzzle	Count correct responses	Conditioned Reinforcement and food
16/2	Häderer et al. (2016) [33]	France	Peer-reviewed paper	Fish (*Tripterygion tripteronotum*)	Mixed	Not clearly stated	Not applicable	Natural environment/home range	Group design	Target black/white chip with muzzle	Count correct responses	Food only
17/1	Hendriksen et al. (2011) [10]	Denmark	Peer-reviewed paper	Horse (*Equus caballus*)	Mixed	7–20 years	Clicker	Stable	Group design	Stepping into trailer on cue	Time to criterion	Conditioned Reinforcement and food
17/2	Hendriksen et al. (2011) [10]	Denmark	Peer-reviewed paper	Horse (*Equus caballus*)	Mixed	7–20 years	Not applicable	Stable	Group design	Stepping into trailer on cue	Time to criterion	Negative reinforcement
18/1	Langbein et al. (2007) [51]	Germany	Peer-reviewed paper	Goat (*Capra hircus*)	Male intact	≤12 months	Beep	Stable	Group design	Shape discrimination	Number of sessions to completion	Conditioned Reinforcement and water
18/2	Langbein et al. (2007) [51]	Germany	Peer-reviewed paper	Goat (*Capra hircus*)	Male intact	≤12 months	Not applicable	Stable	Group design	Shape discrimination	Number of sessions to completion	Control (water only)
19/1	Lansade et al. (2018) [24]	France	Peer-reviewed paper	Horse (*Equus caballus*)	>50% female intact	≤12 months	Spoken word	Stable	Group design	Nose touch cone	Count correct responses	Conditioned Reinforcement and food
19/2	Lansade et al. (2018) [24]	France	Peer-reviewed paper	Horse (*Equus caballus*)	>50% female intact	≤12 months	Not applicable	Stable	Group design	Nose touch cone	Count correct responses	Food only
20/1	McCall et al. (2002) [25]	USA	Peer-reviewed paper	Horse (*Equus caballus*)	Not clearly stated	6 months–15 years	Buzzing sound	Stable	Group design	Pushing a lever	Mean training time	Conditioned Reinforcement and food (Phase I)
20/2	McCall et al. (2002) [25]	USA	Peer-reviewed paper	Horse (*Equus caballus*)	Not clearly stated	6 months–15 years	Not applicable	Stable	Group design	Pushing a lever	Mean training time	Food only (Phase I)
21/1	Meyer et al. (2008) [74]	Denmark	Peer-reviewed paper	Dog (*Canis familiaris*)	50% female	1–5 years	Clicker	Laboratory	Group design	Paw target mouse pad	Number of sessions to completion	Treatment intensities
21/2	Meyer et al. (2008) [74]	Denmark	Peer-reviewed paper	Dog (*Canis familiaris*)	50% female	1–5 years	Clicker	Laboratory	Group design	Paw target mouse pad	Number of sessions to completion	Treatment intensities
**22/1**	**Pfaller-Sadovsky et al. (2017)** [8]	**Austria**	**Peer-reviewed paper**	**Dog (*Canis familiaris*)**	**50% female**	**1–9 years**	**Clicker**	**Home**	**Single-case method**	**Delivering PI to hand**	**Percent trials**	**Conditioned Reinforcement and food**
23/1	Smith et al. (2008) [26]	USA	Peer-reviewed paper	Dog (*Canis familiaris*)	Mixed	1–9 years	Clicker	Home	Group design	Nose touch cone	Latency	Conditioned Reinforcement and food
23/2	Smith et al. (2008) [26]	USA	Peer-reviewed paper	Dog (*Canis familiaris*)	Mixed	1–9 years	Clicker	Home	Group design	Nose touch cone	Latency	Conditioned Reinforcement and INT food
23/3	Smith et al. (2008) [26]	USA	Peer-reviewed paper	Dog (*Canis familiaris*)	Mixed	1–9 years	Not applicable	Home	Group design	Nose touch cone	Latency	Control (food only)
24/1	Strychalski et al. (2015) [75]	Poland	Peer-reviewed paper	Dog (*Canis familiaris*)	50% female	>4 years	Clicker	Home	Group design	Around cones	Number of sessions to completion	Conditioned Reinforcement and food
25/1	Thorn et al. (2006) [76]	USA	Peer-reviewed paper	Dog (*Canis familiaris*)	50% female	Not clearly stated	Spoken word	Shelter room	Case study	Sit (Experiment 1)	Latency	Conditioned Reinforcement and food
25/2	Thorn et al. (2006) [76]	USA	Peer-reviewed paper	Dog (*Canis familiaris*)	50% female	Not clearly stated	Spoken word	Outdoor enclosure	Group design	Sit (Experiment 2)	Latency	Conditioned Reinforcement and food
25/3	Thorn et al. (2006) [76]	USA	Peer-reviewed paper	Dog (*Canis familiaris*)	50% female	Not clearly stated	Clicker	Outdoor enclosure	Group design	Sit (Experiment 2)	Latency	Conditioned Reinforcement and food
**26/1**	**Wennmacher (2007) [27]**	**USA**	**Master’s thesis**	**Dog (*Canis familiaris*)**	**Mixed**	**1–5 years**	**Clicker**	**Home**	**Single-case method**	**Spin, bow**	**Count correct responses**	**Conditioned Reinforcement and food**
**26/2**	**Wennmacher (2007) [27]**	**USA**	**Master’s thesis**	**Dog (*Canis familiaris*)**	**Mixed**	**1–5 years**	**Clicker**	**Home**	**Single-case method**	**Spin, bow**	**Count correct responses**	**Conditioned Reinforcement and INT food**
27/1	Whistance et al. (2009) [77]	UK	Peer-reviewed paper	Cattle (*Bos taurus*)	Female intact	12–24 years	Clicker	Stable	Case study	Eliminate in designated area	Count correct responses	Conditioned Reinforcement and food
28/1	Williams et al. (2004) [34]	USA	Peer-reviewed paper	Horse (*Equus caballus*)	>50% male neutered	>4 years	Clicker	Stable	Group design	Nose touch cone	Number of sessions to completion	Conditioned Reinforcement and food
28/2	Williams et al. (2004) [34]	USA	Peer-reviewed paper	Horse (*Equus caballus*)	>50% male neutered	>4 years	Clicker	Stable	Group design	Nose touch cone	Number of sessions to completion	Conditioned Reinforcement and INT food
28/3	Williams et al. (2004) [34]	USA	Peer-reviewed paper	Horse (*Equus caballus*)	>50% male neutered	>4 years	Not applicable	Stable	Group design	Nose touch cone	Number of sessions to completion	Food only
29/1	Willis et al. (2004) [78]	UK	Peer-reviewed paper	Dog (*Canis familiaris*)	Mixed	Not clearly stated	Clicker	Training facility	Case study	Scent discrimination (healthy/ill)	Count correct responses	Conditioned Reinforcement and food
30/1	Willson et al. (2017) [28]	New Zealand	Peer-reviewed paper	Cat (*Cattus syslvestris*)	>50% male neutered	6 months–15 years	Beep	Shelter room	Group design	Nose touching a target	Shaping steps completed	Conditioned Reinforcement and food
30/2	Willson et al. (2017) [28]	New Zealand	Peer-reviewed paper	Cat (*Cattus syslvestris*)	>50% male neutered	6 months–15 years	Not applicable	Shelter room	Group design	Nose touching a target	Shaping steps completed	Food only
30/3	Willson et al. (2017) [28]	New Zealand	Peer-reviewed paper	Cat (*Cattus syslvestris*)	>50% male neutered	6 months–15 years	Beep	Shelter room	Group design	Nose touching a target	Number of attempts over time	Conditioned Reinforcement only
30/4	Willson et al. (2017) [28]	New Zealand	Peer-reviewed paper	Cat (*Cattus syslvestris*)	>50% male neutered	6 months–15 years	Beep	Shelter room	Group design	Nose touching a target	Number of attempts over time	Conditioned Reinforcement only
31/1	Wood (2007) [29]	USA	Master’s thesis	Dog (*Canis familiaris*)	>50% male neutered	1–5 years	Clicker	Shelter room	Group design	Nose touching a freestanding target	Required Rs to reach criterion	Conditioned Reinforcement and food
31/2	Wood (2007) [29]	USA	Master’s thesis	Dog (*Canis familiaris*)	>50% male neutered	1–5 years	Spoken word	Shelter room	Group design	Nose touching a freestanding target	Required Rs to reach criterion	Conditioned Reinforcement and food
**32/1**	**Rybova (2018)** [23]	**New Zealand**	**Master’s thesis**	**Horse (*Equus caballus*)**	**50% female**	**7–28 years**	**Clicker**	**Stable**	**Single-case method**	**Nose-touch target stick**	**Count correct responses**	**Conditioned Reinforcement and food**
33/1	Martinez de Andino et al. (2017) [50]	USA	Peer-reviewed paper	Horse (*Equus caballus*)	50% female	≤6 months	Spoken word	Outdoor enclosure	Group design	Touching a floor target with nose (i.e., stone)	Latency	Conditioned Reinforcement and tactile
33/2	Martinez de Andino et al. (2017) [50]	USA	Peer-reviewed paper	Horse (*Equus caballus*)	50% female	≤6 months	Spoken word	Outdoor enclosure	Group design	Touching a floor target with nose (i.e., stone)	Count correct responses	Conditioned Reinforcement and tactile
33/3	Martinez de Andino et al. (2017) [50]	USA	Peer-reviewed paper	Horse (*Equus caballus*)	50% female	≤6 months	Spoken word	Outdoor enclosure	Group design	Touching a floor target with nose (i.e., stone)	Correct response to verbal prompt rate	Conditioned Reinforcement and tactile
33/4	Martinez de Andino et al. (2017) [50]	USA	Peer-reviewed paper	Horse (*Equus caballus*)	50% female	≤6 months	Spoken word	Outdoor enclosure	Group design	Touching a floor target with nose (i.e., stone)	Latency	Conditioned Reinforcement and tactile
33/5	Martinez de Andino et al. (2017) [50]	USA	Peer-reviewed paper	Horse (*Equus caballus*)	50% female	≤6 months	Spoken word	Outdoor enclosure	Group design	Touching a floor target with nose (i.e., stone)	Count correct responses	Conditioned Reinforcement and tactile
33/6	Martinez de Andino et al. (2017) [50]	USA	Peer-reviewed paper	Horse (*Equus caballus*)	50% female	≤6 months	Spoken word	Outdoor enclosure	Group design	Touching a floor target with nose (i.e., stone)	Correct response to verbal prompt rate	Conditioned Reinforcement and tactile
**34/1**	**Slater et al. (2011) [9]**	**UK**	**Peer-reviewed paper**	**Horse (*Equus caballus*)**	**Male neutered**	**5–18 years**	**Clicker**	**Outdoor enclosure**	**Single-case method**	**Loading trailer**	**Loading steps completed**	**Conditioned Reinforcement and food**
**34/2**	**Slater et al. (2011) [9]**	**UK**	**Peer-reviewed paper**	**Horse (*Equus caballus*)**	**Male neutered**	**5–18 years**	**Clicker**	**Stable**	**Single-case method**	**Lifting hoof**	**Duration**	**Conditioned Reinforcement and INT food**

**Table 3:** Count and percentage of studies by species and type of conditioned reinforcers (S^r+^), across types of unconditioned reinforcers (S^R+^).

Learner Species	Type of S^r+^	Food								Scratching		Water		No S^R+^		Not clearly Stated		Total	
		By Hand		Remotely		Container		Proximity											
		Count	%	Count	%	Count	%	Count	%	Count	%	Count	%	Count	%	Count	%	Count	%
Cat (*Cattus syslvestris*)	Beep	0	0%	1	3%	0	0%	0	0%	0	0%	0	0%	0	0%	0	0%	1	3%
	Clicker	1	3%	0	0%	0	0%	0	0%	0	0%	0	0%	0	0%	0	0%	1	3%
Cattle (*Bos taurus*)	Clicker	1	3%	0	0%	0	0%	0	0%	0	0%	0	0%	0	0%	0	0%	1	3%
Dog (*Canis familiaris*)	Clicker	11	32%	0	0%	1	3%	1	3%	0	0%	0	0%	0	0%	1	3%	14	41%
	Spoken word	1	3%	0	0%	0	0%	0	0%	0	0%	0	0%	0	0%	0	0%	1	3%
	Whistle	0	0%	0	0%	0	0%	1	3%	0	0%	0	0%	0	0%	0	0%	1	3%
Fish (*Tripterygion tripteronotum*)	Clicker	0	0%	0	0%	1	3%	0	0%	0	0%	0	0%	0	0%	0	0%	1	3%
Goat (*Capra hircus*)	Beep	0	0%	0	0%	0	0%	0	0%	0	0%	1	3%	0	0%	0	0%	1	3%
	Clicker	1	3%	0	0%	0	0%	0	0%	0	0%	0	0%	0	0%	0	0%	1	3%
Horse (*Equus caballus*)	Buzzing sound	0	0%	0	0%	1	3%	0	0%	0	0%	0	0%	0	0%	0	0%	1	3%
	Clicker	5	15%	0	0%	2	6%	0	0%	0	0	0	0%	0	0%	0	0%	7	21%
	Spoken word	0	0%	0	0%	0	0%	0	0%	1	3%	0	0%	1	3%	0	0%	2	6%
Rhesus monkey (*Macaca mulatta*)	Clicker	1	3%	0	0%	0	0%	0	0%	0	0%	0	0%	0	0%	0	0%	1	3%
Squirrel monkey (*Saimiri boliviensis*)	Clicker	1	3%	0	0%	0	0%	0	0%	0	0%	0	0%	0	0%	0	0%	1	3%
**Total**		**22**	**65%**	**1**	**3%**	**5**	**15%**	**2**	**6%**	**1**	**3%**	**1**	**3%**	**1**	**3%**	**1**	**3%**	**34**	**100%**

Note: For the case of food (S^R+^), the mode of delivery is specified (i.e., by hand, by remotely controlled feeder, presented in a container, or delivered in proximity), see Table 1 for details. Percentages may not total 100% due to rounding.

**Table 4:** Implementation of preference assessment displayed by type of unconditioned reinforcers (S^R+^).

Type of S^R+^		Preference Assessment	Count	%
Food	Food by hand	No	20	59%
		Yes	1	3%
		Not clearly stated	1	3%
	Food remotely	No	1	3%
	Food container	No	5	15%
	Food proximity	No	1	3%
		Not clearly stated	1	3%
Scratching		Yes	1	3%
Water		Not applicable	1	3%
No S^R+^		Not applicable	1	3%
Not clearly stated		Not clearly stated	1	3%
**Total**			**34**	**100%**

Note: Percentages may not total 100% due to rounding.

**Table 5:** Number of pairings and respective interstimulus intervals (ISI) between conditioned (CS) and unconditioned stimuli (US) reported across eligible studies that reported separate pairings.

CS-US Pairing Information	Interstimulus Interval ISI	Count	%
>20 pairings (no further info)	30 s–3 min	1	3%
	Not clearly stated	1	3%
≤20 pairings/day	Approximately 1 s (trace)	2	6%
	Approximately 10 s	1	3%
	No delay (simultaneous)	1	3%
	Not clearly stated	5	15%
20–40 pairings/day	Immediately	1	3%
	No delay (simultaneous)	1	3%
	Not clearly stated	2	6%
60 pairings/day	Not clearly stated	1	3%
Pairings implemented (no further info)	Immediately	1	3%
	No delay (simultaneous)	1	3%
	Not clearly stated	3	9%
No pairing sessions	Not applicable	10	29%
Not clearly stated	Not clearly stated	3	9%
**Total**		**34**	**100%**

Note: Percentages may not total 100% due to rounding.

**Table 6:** Approximations of the delays between the display of the target responses (R; e.g., nose touch) and the onset of the conditioned reinforcer (S^r+^; e.g., click or spoken word) reported across all eligible studies.

Delay R → S^r+^	Beep		Buzzing Sound		Clicker		Spoken Word		Whistle		Total	
	Count	%	Count	%	Count	%	Count	%	Count	%	Count	%
Approximately 1 s	0	0%	0	0%	1	3%	0	0%	0	0%	1	3%
Immediately	0	0%	0	0%	3	9%	1	3%	0	0%	4	12%
Immediately after	0	0%	0	0%	1	3%	0	0%	0	0%	1	3%
Simultaneous	0	0%	0	0%	3	9%	1	3%	0	0%	4	12%
Shortly after	0	0%	0	0%	1	3%	0	0%	0	0%	1	3%
Not clearly stated	2	6%	1	3%	18	53%	1	3%	1	3%	23	68%
**Total**	**2**	**6%**	**1**	**3%**	**27**	**79%**	**3**	**9%**	**1**	**3%**	**34**	**100%**

Note: Percentages may not total 100% due to rounding.

**Table 7:** Information extracted from the reviewed studies on the interval between the conditioned reinforcer (S^r+^) and the delivery of the unconditioned reinforcement (S^R+^) reported across all eligible studies.

Interval S^r+^ → S^R+^	Food								Scratching		Water		No S^R+^		Not Clearly Stated		Total	
	Food by Hand		Food Remotely		Food Container		Food Proximity											
	Count	%	Count	%	Count	%	Count	%	Count	%	Count	%	Count	%	Count	%	Count	%
Approximately 1 s	1	3%	0	0%	1	3%	0	0%	0	0%	0	0%	0	0%	0	0%	2	6%
Following	2	6%	0	0%	1	3%	0	0%	0	0%	0	0%	0	0%	0	0%	3	9%
Immediately	3	9%	0	0%	1	3%	1	3%	0	0%	0	0%	0	0%	0	0%	5	15%
Shortly after	1	3%	0	0%	0	0%	0	0%	0	0%	0	0%	0	0%	0	0%	1	3%
No delay (simultaneous)	0	0%	0	0%	0	0%	0	0%	1	3%	1	3%	0	0%	0	0%	2	6%
Not clearly stated	15	44%	1	3%	2	6%	1	3%	0	0%	0	0%	0	0%	1	3%	20	59%
Not applicable *	0	0%	0	0%	0	0%	0	0%	0	0%	0	0%	1	3%	0	0%	1	3%
**Total**	**22**	**65%**	**1**	**3%**	**5**	**15%**	**2**	**6%**	**1**	**3%**	**1**	**3%**	**1**	**3%**	**1**	**3%**	**34**	**100%**

Note: Percentages may not total 100% due to rounding. * = the S^r+^ was not followed by the S^R+^.

**Table 8:** Positioning of experimenters and trainers with respective reinforcers displayed by learner species.

Learner Species	In Front, S^R+^ Attached		In Front, S^R+^ Proximity		In Front, No S^R+^		Outside View, S^R+^ Proximity		Peripheral View, S^R+^ Attached		Peripheral View, S^R+^ Proximity		Not Applicable		Not Clearly Stated		Total	
	Count	%	Count	%	Count	%	Count	%	Count	%	Count	%	Count	%	Count	%	Count	%
Cat (*Cattus syslvestris*)	0	0%	0	0%	0	0%	0	0%	0	0%	0	0%	0	0%	2	6%	2	6%
Cattle (*Bos taurus*)	0	0%	0	0%	0	0%	0	0%	0	0%	0	0%	0	0%	1	3%	1	3%
Dog (*Canis familiaris*)	8	24%	0	0%	0	0%	0	0%	0	0%	0	0%	0	0%	8	24%	16	47%
Fish (*Tripterygion tripteronotum*)	0	0%	1	3%	0	0%	0	0%	0	0%	0	0%	0	0%	0	0%	1	3%
Goat (*Capra hircus*)	0	0%	0	0%	0	0%	0	0%	0	0%	0	0%	1	3%	1	3%	2	6%
Horse (*Equus caballus*)	0	0%	1	3%	1	3%	1	3%	1	3%	1	3%	0	0%	5	15%	10	29%
Rhesus monkey (*Macaca mulatta*)	0	0%	0	0%	0	0%	0	0%	0	0%	0	0%	0	0%	1	3%	1	3%
Squirrel monkey (*Saimiri boliviensis*)	0	0%	1	3%	0	0%	0	0%	0	0%	0	0%	0	0%	0	0%	1	3%
**Total**	**8**	**24%**	**3**	**9%**	**1**	**3%**	**1**	**3%**	**1**	**3%**	**1**	**3%**	**1**	**3%**	**18**	**53%**	**34**	**100%**

Note: Percentages may not total 100% due to rounding.

**Table 9:** Study characteristics and effect sizes per study.

Author (Year)	Species	#Subjects	Design	Reinforcers	Tau-U (CI_95%_)	Contrasts	Effect	Design Quality
Ferguson and Rosales-Ruiz (2001) [21]	Horse	5	MBL	Click and food	0.95 (0.78, 1)	15	Large	Strong
Fernandez * (2003) [4]	Goat	3	CCD	Click and food	0.51 (0.3, 0.71)	6	Small	Strong
Pfaller-Sadovsky et al. (2017) [8]	Dog	4	MBL	Click and food	0.77 (0.49, 1)	7	Medium	Strong
Rybova (2018) * [30]	Horse	4	MBL	Click and food	0.90 (0.59, 1)	8	Medium	Moderate
Wennmacher * (2007) [25]	Dog	2	RVD	Click and food versus Click-click food	0.48 (0.33, 0.61)	12	Small	Moderate
Slater and Dymond (2011) [9]	Horse	5	MBL	Click and food	0.98 (0.73, 1)	4	Large	Strong

Note: CCD = changing criterion design; CI = confidence interval; MBL = multiple-baseline design; RVD = reversal design. * = Master’s theses.

**Table 10:** Effect sizes and standard errors by potential moderators.

Moderator Variables	Number of Covariates	Regression ES and (SE)	*Q*	*p*-Values	R^2^ (%)
Learner species	3		20.57	0.000 *	1
Dogs		0.573 (0.067)			
Goats		0.574 (0.11)			
Horses		0.94 (0.09)			
Pairing sessions	4		1.42	0.70	0
>20 pairing sessions		0.94 (0.23)			
20–40 pairings/day		0.76 (0.28)			
60 pairings/day		0.90 (0.35)			
No pairings		0.63 (0.29)			
**Position of trainer with S^r+^**	3		1.28	0.53	0
**In front of animal w/S^r+^ attached**		0.63 (0.16)			
Not clearly stated		0.82 (0.20)			
**Within peripheral view w/S^r+^ attached**		0.90 (0.29)			
Study design	3		26.86	0.0001 *	1
MBL		0.92 (0.06)			
Changing criterion		0.34 (0.10)			
Reversal/withdrawal		0.44 (0.09)			
Target behavior by	4		2.59	0.46	12
Capturing w/movement restrictions		0.57 (0.20)			
**Capturing w/o movement restrictions**		0.94 (0.28)			
Shaping w/prompts		0.67 (0.25)			
Shaping w/o prompts		0.88 (0.26)			
Trainer type	3		0.11	0.94	0
Experimenter		0.80 (0.15)			
Mixed		0.72 (0.25)			
Owner		0.78 (0.32)			

Note: ES = effect size; SE = standard error; statistically significant results are highlighted with an asterisk.
